# Time for change: compliance with RCS green theatre checklist—facilitators and barriers on the journey to net zero

**DOI:** 10.3389/fsurg.2023.1260301

**Published:** 2023-10-24

**Authors:** Elizabeth Westwood, Josephine Walshaw, Katie Boag, WeiYing Chua, Safaa Dimashki, Hammaad Khalid, Ross Lathan, Jack Wellington, Sonia Lockwood, Marina Yiasemidou

**Affiliations:** ^1^ General Surgery, Bradford Teaching Hospitals NHS Foundation Trust, Bradford, United Kingdom; ^2^ Department of Health Research, University of York, York, United Kingdom; ^3^ General Surgery, Hull University Teaching Hospitals NHS Trust, Hull, United Kingdom; ^4^ Clinical Sciences Centre, Hull York Medical School, Hull, United Kingdom; ^5^ Leeds Institute of Medical Research, St. James's University Hospital, University of Leeds, Leeds, United Kingdom; ^6^ School of Medicine, University of Nottingham, Nottingham, United Kingdom; ^7^ Academic Unit of Vascular Surgery, Hull University Teaching Hospitals NHS Trust, Hull, United Kingdom; ^8^ School of Medicine, Cardiff University, Cardiff, United Kingdom; ^9^ Colorectal Surgery, Oxford University Hospitals NHS Foundation Trust, Oxford, United Kingdom

**Keywords:** climate change, surgery, sustainability, green, theatre

## Abstract

**Background:**

Climate change is an era-defining health concern, with healthcare related emissions paradoxically compounding negative impacts. The NHS produces 5% of the UK's carbon footprint, with operating theatres a recognised carbon hotspot. NHS England aims to become Net Zero by 2045. Consequently, UK Royal Colleges of Surgery have published guidance to foster an evidence-based sustainable transformation in surgical practice.

**Methods:**

A single-centre quality improvement project was undertaken, aiming to provide an overview of sustainable practice locally. The Intercollegiate “Green Theatre Checklist” was taken as an audit standard, focusing on “preparing for surgery” and “intraoperative equipment” subsections. Any general surgical procedure was eligible for inclusion. Usage of reusable textiles, non-sterile gloves, catheters, antibiotics, alcohol vs. water-based scrub techniques, skin sterilisation choices, and skin closure materials were recorded. Baseline data collection occurred over a 3 week period, followed by dissemination of results locally via clinical governance meetings and poster displays. A re-audit of practice was conducted using the same methodology and duration.

**Results:**

Datasets 1 (*n* = 23) and 2 (*n* = 23) included open (*n* = 22), laparoscopic (*n* = 24), elective (*n* = 22) and non-elective (*n* = 24) cases. Good practice was demonstrated in reusable textiles (trolley covers 96%, 78%, drapes 100%, 92%) however procurement issues reduced otherwise good reusable gown use in Dataset 2 in (90%, 46%). No unnecessary catheter use was identified, and loose skin preparations were used unanimously. Uptake of alcohol-based scrubbing techniques was low (15%, 17%) and unnecessary non-sterile glove use was observed in >30% of procedures. All laparoscopic ports and scissors were single use. Carbon footprints were 128.27 kgCO2e and 117.71 kgCO2e in datasets 1 and 2 respectively.

**Conclusion:**

This project evidences good practice alongside future local focus areas for improved sustainability. Adoption of hybrid laparoscopic instruments, avoiding unnecessary equipment opening, and standardising reusable materials could reduce carbon and environmental impact considerably. Successful implementation requires considered procurement practices, improved awareness and education, clear leadership, and a sustained cultural shift within the healthcare community. Collaboration among professional institutions and access to supporting evidence is crucial in driving engagement and empowering clinicians to make locally relevant changes a reality.

## Introduction

Climate change is an era-defining concern, with varied and profoundly negative impacts (1). At 1.1°C of warming from pre-industrial averages, we are already witnessing the direct and immediate effects of this upward trend, including more frequent and severe weather events, increased morbidity and mortality across various health outcomes, and higher rates of vector-borne diseases (2). Ongoing global dependency on fossil fuel consumption is likely to see such trends continue, with existing policies putting the world on track for a 2.4–3.5°C rise by 2,100, far exceeding the 1.5°C target set by the Paris Agreement in 2015 (2).

Heath services contribute to 4%–5% of global greenhouse gas emissions. This is predominantly carbon dioxide, along with nitrous oxide, methane, and anaesthetic gases (1). Recognising the urgency of the situation, the United Nations Climate Change Conference (COP26) in 2021 outlined initiatives on climate-resilient and sustainable low-carbon health systems (3). Fifty countries committed to this action plan, with fourteen countries setting targets for achieving net zero emissions by 2050 (4). In line with these efforts, UK National Health Service (NHS) aims to achieve Net Zero emissions for both direct and indirect sources by 2045 through reducing the carbon footprint of healthcare services and promoting sustainable practices across all areas, including surgical settings (5). These targets, combined with the concerted efforts of healthcare professionals, policymakers, and researchers, demonstrate a collective commitment to driving positive change and promoting sustainability within the healthcare sector (6).

In response to these challenges, healthcare institutions are increasingly adopting sustainable practices to minimise their environmental impact. Operating theatres in particular are recognised as carbon and resource-intensive areas within hospital settings, contributing to 25% of carbon emissions despite less than 5% of inpatients undergoing surgery (7). To address this issue, the collaborative “Intercollegiate Green Theatre Checklist” has been developed, offering evidence-based guidelines for sustainable practice in surgical settings, and serving as an established benchmark for improving practice (8).

The aim of this quality improvement (QI) project is to comprehensively assess and implement sustainable theatre practices in a surgical setting, utilising strategies based on the “Green Theatre Checklist” to align with national targets. We will also use the Life Cycle Assessment (LCA) approach to map carbon emissions to evaluate the environmental impact of our interventions. The findings of this project will not only provide insights into the current state of sustainable practices in this clinical setting, but also offer valuable recommendations for healthcare institutions seeking to implement similar initiatives.

## Methods

This initiative received local approval by the clinical effectiveness team at Bradford Teaching Hospitals. The framework of this article is reported in accordance with Revised Standards for Quality Improvement Reporting Excellence (SQUIRE 2.0) (9).

### Context

This single-centre QI project was undertaken in the Department of General Surgery at Bradford Royal Infirmary, Bradford Teaching Hospitals NHS Foundation Trust, UK. This busy teaching hospital surgery department provides Colorectal, Upper Gastrointestinal, and Emergency General Surgery to serve a population of around 500,000 people from the surrounding area (10).

The Intercollegiate “Green Theatre Checklist”, collaboratively published by the Royal College of Surgeons of Edinburgh, Royal College of Surgeons of England, Royal College of Surgeons of Ireland and Royal College of Physicians and Surgeons of Glasgow, was taken as an audit standard. Outcomes based on the “preparing for surgery” and “intraoperative equipment” subsections were chosen. This decision was driven by a desire to look most closely at areas of influence and importance for clinical members of the operating team specifically.

A bespoke data collection form was created on Google Forms (Supplementary Appendix S1). No patient identifiable information was gathered. Demographic parameters for each recorded procedure included the responsible consultant surgeon, acuity (emergency, sub-acute or elective), open vs. laparoscopic methods, procedure title/description and the number of scrubbed staff members within the sterile field. A QR code linking to the data collection form was disseminated to surgical trainees and use was encouraged during the data collection period. Baseline data was collected during a 3 week period between February and March 2023 prospectively by surgical trainees participating in each of the procedures.

### Interventions

The results obtained from the initial data collection period were analysed and disseminated to the department during the local clinical governance meeting. As part of this process, education was provided to raise awareness about the environmental impact of the operating theatre. Additionally, posters illustrating the environmental impacts and promoting positive behaviour changes, in accordance with the recommendations outlined by the “Green Theatre Checklist”, were prominently displayed in the General Surgery theatres (Supplementary Appendix S2). Following these interventions, a re-audit of practice was conducted over a 3 week period in May and June 2023 using the same method as the pre-intervention data collection period.

### Sustainability criteria

Measured sustainability criteria included the number of reusable and disposable textiles (gowns, hats, trolley covers and drapes), number of staff performing alcohol based scrubbing techniques (as opposed to water/soap based techniques), catheter use, antibiotic use, use of reusable and disposable kidney dishes, choice of skin sterilisation method, choice of skin closure materials, observation of un-necessary glove use, use of sterile gowns around the theatre when not a performing a sterile task, and opening/disposal of unused equipment.

### Carbon emission analysis

A LCA approach was used to map greenhouse gas emissions, in line with ISO 14,067 Guidelines (11) (emissions were reported as kilogram carbon dioxide equivalent (kgCO2e). Wherever possible, carbon footprint estimates were based on data from published, comprehensive life-cycle analyses, using bottom up methodology from UK based, up to date datasets (12–14). These estimates account for raw material manufacture, use, transport, associated packaging, laundering/sterilisation processes in the case of reusable items and eventual disposal. Life-cycle estimates were possible for most textiles and surgical equipment with the exception of trolley covers, scrubbing soaps, specialised equipment (e.g., purse string clamp) and antibiotics. For some of these items financial proxies have been used to produce top down figures. Alternative comparisons based on reduction or increase in resource use without specific carbon quantification have also been used where relevant e.g., % change in observations of unnecessary non-sterile glove use or water usage in litres resulting from water based scrubbing (15).

### Assumptions and definitions

For elective and sub-acute cases, it was assumed that when disposable hats were worn, each staff member wore the same hat throughout a given ½ day operating session. For emergency cases it was assumed that a new disposable hat was donned for each new case. Where reusable garments such as hats and gowns are used, estimated lifespan was 75 uses—an average derived by life-cycle analysis source data via direct discussions with manufacturers. In the case of hats each “use” could account for up to 4 operations as it was assumed that hats were laundered on average after this many cases. The carbon footprint of reusables becomes smaller with increased uses over their lifespan.

Inappropriate non-sterile glove use was defined as glove use in the absence of potential contact with bodily fluid, mucous membranes, non-intact skin or specific infection control measures.

The use of alcohol based scrubbing techniques were deemed appropriate when being performed after at least one prior thorough water/soap based scrub, as per NICE guidance (16).

The average water consumption per water/soap based scrub was 18.5l (15) and carbon footprint of 1 litre of water was taken to be 0.00136927 kgCO2e (17).

Whilst variation in practice based on patient specific factors exists, for the purposes of this study indications for appropriate antibiotic use were a) the use of surgical implants or b) surgery on a contaminated site (16).

## Results

Overall, 46 surgical procedures were assessed. Baseline data from 23 procedures, overseen by 8 consultants, with a mix of elective (*n* = 16) and non-elective (*n* = 7) General Surgical caseload were recorded over the initial period of 3 weeks in February and March 2023. There were 13 procedures, involving a total 53 scrubbed staff members, during which it would have been clinically appropriate to choose an alcohol-based scrubbing technique—i.e., not the first procedure of the day/session. The mean number of staff scrubbed per case over all 23 procedures was 4 (range 3–6).

Following education and poster displays, a further 23 procedures overseen by 7 consultants with a mix of elective (*n* = 6), non-elective (*n* = 17) General Surgical caseload were recorded over a period of 3 weeks in May and June 2023. There were 12 procedures involving 35 scrubbed staff members during which it would have been clinically appropriate to choose an alcohol base scrubbing technique—i.e., not the first procedure of the day/session. The mean number of staff scrubbed per case over all 23 procedures was 3 (range 2–5).

We summarised the operation characteristics for each data collection period in Table 1.

**Table 1:** Procedure descriptions arranged according to surgical approach (laparoscopic vs. open).

	Dataset 1 (*n*)	Dataset 2 (*n*)	Totals (*n*)
Laparoscopic procedures			
Laparoscopic cholecystectomy	8	1	9
Laparoscopic appendicectomy	2	2	4
Laparoscopic sleeve gastrectomy and cholecystectomy	1	0	1
Laparoscopic nissen fundoplication	1	0	1
Laparoscopic subtotal colectomy	1	0	1
Laparoscopic giant hiatus hernia repair	1	0	1
Laparoscopic inguinal hernia repair	2	0	2
Laparoscopic adhesiolysis	0	1	1
Laparoscopic converted to open bowel resection with ileostomy	0	1	1
Laparoscopic high anterior resection	0	1	1
Totals (laparoscopic)	16	6	22
Open procedures			
Epigastric hernia repair	1	0	1
Incisional hernia repair	1	0	1
Peristomal hernia repair	1	0	1
Open inguinal hernia repair	0	1	1
Umbilical hernia repair	0	1	1
Incision and drainage of abscess	0	8	8
Laparotomy and right hemicolectomy with anastomosis	0	1	1
Laparotomy and small bowel bypass	0	1	1
Laparotomy and adhesiolysis	0	1	1
EUA umbilicus and toilet	1	0	1
EUA and banding of haemorrhoids	1	0	1
EUA anorectum and manual disimpaction	0	1	1
EUA anorectum, abscess drainage and insertion of seton	0	1	1
Left groin lymph node dissection	0	1	1
Excision of papillomas	0	1	1
Reversal loop ileostomy	1	0	1
Total Gastrectomy	1	0	1
Totals (Open)	7	17	24
Totals (laparoscopic and open)	23	23	46

## Section 7: reusable textiles

### Hats

A total of 106 hats were used in the first data collection period, of which 2 (2%) were re-usable. During the second data collection period 101 hats were used, *n* = 18 (18%) of which were re-usable. The carbon footprint of 1 disposable hat was estimated to be 0.00354 kgCO2e, whilst a reusable equivalent was 0.00366 kgCO2e. The carbon footprint for hats during data collection period 1 was 0.38 kgCO2e, and during data collection period 2 was 0.35 kgCO2e—this amounted to a 0.021 kgCOe reduction.

### Gowns

Reusable gowns accounted for 90% (*n* = 84) of gowns used in the first data collection window, however this fell to 46% (*n* = 33) in the second. A reusable gown was estimated to have a carbon footprint of 0.253 kgCO2e per use compared to 0.649 kgCO2e for its disposable equivalent. The footprint from gowns in dataset 1 was 27.04 kgCO2e compared with 32.97 kgCO2e in dataset 2. Despite using 22 fewer gowns in total during the second data collection window, carbon footprint increased by 5.93 kgCO2e as a result of the higher proportion of disposables in use.

As well as gowns used within the sterile field, sterile gowns are sometimes donned informally outside this field as an extra clothing layer. In dataset 1, *n* = 9 gowns (8 reusable (2.02 kgCo2e), 1 disposable (0.65 kgCO2e)) were noted to be used in this way. In dataset 2, *n* = 11 gowns (7 reusable (1.77 kgCO2e), 4 disposable (2.60 kgCO2e)) were noted to be used in this way. Elimination of this practice would save 6.44 kgCO2e collectively.

### Drapes

Reusable drapes were invariably used in the first data collection period (100%, 3.20 kgCO2e total), whilst in the second, a disposable drape was used in one procedure (*n* = 1) and a combination of disposable and reusable drapes were used in another (*n* = 1) with reusable drapes still being used in the majority of instances (91%) giving a total carbon footprint of 5.50 kgCO2e. This amounts to a net increase of 2.30 kgCO2e in the second data collection period.

### Trolley covers

In the first data collection period 96% of cases (*n* = 22) made use of reusable trolley covers compared with 78% (*n* = 18) in the second. The carbon footprint of a disposable trolley cover was 0.740 kgCO2e. There was insufficient data for the calculation of reusable trolley covers.

## Section 8: reduce water consumption and energy consumption

Overall water consumption was 1573l (2.12 kgCO2e) and 1203l (1.64 kgCO2e) in data collection windows 1 and 2 respectively. Uptake of the alcohol scrubbing techniques for eligible cases was 15% (8/53) during dataset 1% and 17% (6/35) during dataset 2, equating to 148l (0.2 kgCO2e) and 111l (0.15 kgCO2e) water and carbon savings respectively.

## Section 9: avoiding clinically unnecessary interventions

### Antibiotics

In both the first and second data collection windows there were *n* = 2 instances of antibiotics used without clear clinical indication, representing 20% (2/10) and 22% (2/9) of total antibiotic use respectively. The carbon footprint for antibiotic use in data collection periods 1 and 2 were 10.85 kgCO2e and 9.77 kgCO2e respectively. Elimination of non-indicated antibiotic use would reduce these totals by 2.17 kgCO2e each.

### Catheters

13% (*n* = 3) of patients were catheterised for surgery in data collection period 1. This included patients undergoing laparoscopic subtotal colectomy, parastomal hernia repair and total gastrectomy. 17% of patients were catheterised for surgery in data collection period 2. This included patients undergoing laparotomy, right hemicolectomy and anastomosis, laparotomy and small bowel bypass, laparotomy and adhesiolysis and laparoscopic converted to open bowel resection with ileostomy. Catheter usage contributed 11.4 kgCO2e to the surgical carbon footprint of data collection window 1 and 15.2 kgCO2e to data collection period 2. In all cases where catheters were used patients were undergoing procedures of prolonged duration and as such all were deemed to be clinically appropriate.

## Section 10: review and rationalise

In data collection period 1, *n* = 14 sutures (0.25 kgCO2e) were opened but unused. In data collection period 2, *n* = 11 sutures (0.2 kgCO2e), *n* = 1 sorbsan surgical packing ribbon (0.44 kgCO2e), *n* = 2 syringes (0.13 kg CO2e), *n* = 2 needles (0.007 kgCO2e) and *n* = 1 automatic purse string clamp (25.6 kgCO2e) were opened but unused, with a collective carbon footprint of 26.63 kgCO2e.

## Section 11: reduce

Section 11 advises avoidance of unnecessary equipment e.g non-sterile gloves. Observation of unnecessary non-sterile glove use occurred in 34% (*n* = 8) of cases in the first data collection period, and 43% (*n* = 10) of cases in the second. Exact quantification of carbon footprint was not possible given the outcome metric used, however for context a 100 glove box represents 2.6 kgCO2e. Annual glove usage in NHS England and social care for 2020/21 was 5.5 billion (18).

## Section 12: reuse

### Kidney dishes and gallipots

We found that aside from 1 procedure in the first data collection window all kidney dishes and gallipots used were re-usable. Single use, individually packaged kidney dishes are estimated to have a 118 fold greater carbon footprint than reusable alternatives included as part of sterilised surgical trays; 0.073 kgCO2e and 0.00063 kgCO2e respectively. Assuming one kidney dish per case, current practice represents carbon savings of 1.58 kgCO2e and 1.65 kgCO2e over each data collection window compared with using only single use alternatives.

### Laparoscopic equipment

The majority of laparoscopic equipment used was hybrid, however suction catheters, scissors and ports were identified as being single use. We were not able to identify sufficient information to compare suction catheter impact, however footprint estimates for single use scissors (1.14 kgCO2e) and ports (3.50 kgCO2e) amount to 74.14 kgCO2e (*n* = 16 laparoscopic procedures Dataset 1) and 27.80 kgCO2e (*n* = 6 laparoscopic procedures Dataset 2) respectively for current practice. Switching to procurement of hybrid scissors and ports could reduce this impact by 56.36 kgCO2e and 16.74 kgCO2e respectively over each data collection window.

## Section 13: replace

We found that loose skin prep was unanimously used for all procedures in both data collection periods, however there was insufficient data to quantify a comparative carbon footprint.

We found that of the procedures involving skin closure in data collection window 1 (*n* = 21) 48% of these (*n* = 10) were completed using sutures, compared with 54% (*n* = 7) of procedures involving skin closure (*n* = 13) in data collection window 2. The carbon footprint of a stapler (0.37 kgCO2e) is 20 times that of a 3–0 absorbable monofilament suture (0.018 kgCO2e). The carbon footprint of skin closure in data collection window 1 was 4.23 kgCO2e. The carbon of skin closure in data collection window 2 was 2.34 kgCO2e. This is likely an underestimate of the sustainability savings associated with sutures over staplers, as it does not account for carbon, money or patient/staff time embedded in the required return to a healthcare centre for subsequent staple removal.

**Figure 1:**
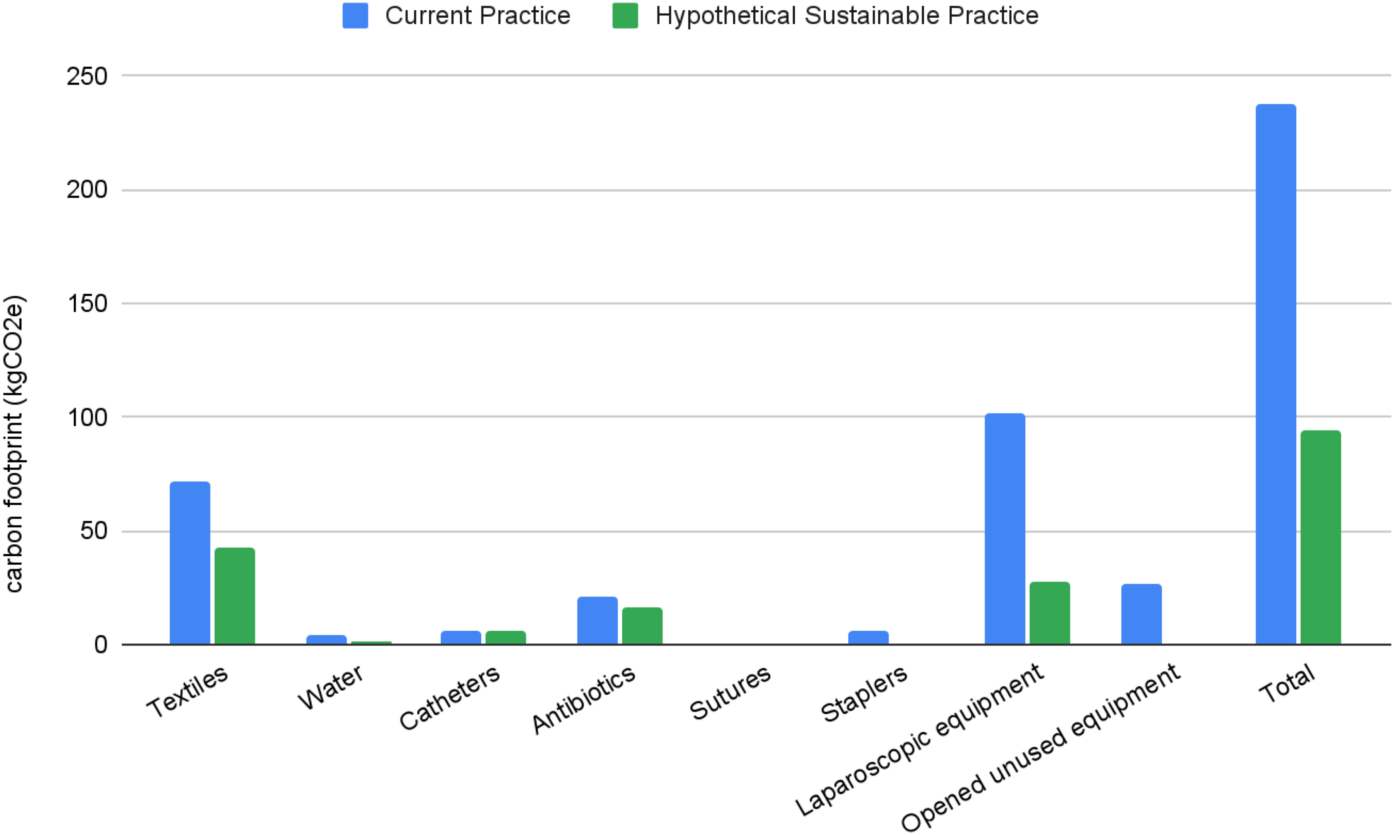
Barchart comparing relative carbon footprint of current practice compared to fully optimised, hypothetical “sustainable practice” scenario over the same time period.

Total carbon footprint for all measured outcomes was 128.27 kgCO2e in dataset 1 and 117.71 kgCO2e in dataset 2 (Table 2). This is equivalent to driving 432 miles and 396 miles respectively in the average petrol car. If we fully optimised all potential sustainable changes currently available (full reusable textile use, full uptake of alcohol-based scrubbing techniques where appropriate, elimination of opened but unused equipment and non-indicated antibiotic use, switching from staplers to sutures and from single use to hybrid laparoscopic equipment) total carbon footprint could be reduced from 246 kgCO2e to 101 kgCO2e (Figure 1).

**Table 2:** Carbon footprints (kgCO2e) according to current practice in datasets 1 and 2, with combined totals over both data collection periods.

	1 item/unit (kgCO2e)	Dataset 1 (kgCO2e)	Dataset 2 (kgCO2e)	Combined dataset total (kgCO2e)
Gowns in sterile field				
Disposable	0.649	5.842	24.668	30.511
Reusable	0.253	21.224	8.338	29.562
Gowns outside sterile field				
Disposable	0.649	0.649	2.597	3.246
Reusable	0.253	2.021	1.169	3.190
Hats				
Disposable	0.004	0.368	0.294	0.662
Reusable	0.004	0.007	0.066	0.073
Drape				
Disposable	1.290	0.000	2.580	2.580
Reusable	0.139	3.197	2.919	6.116
Trolley Cover				
Disposable	0.740	0.740	3.702	4.443
Reusable	–	–	–	–
Water (/L)	0.001	2.154	1.647	3.801
Catheter use	0.863	2.589	3.452	6.041
Antibiotic use (/dose)	1.085	10.846	9.761	20.607
Sutures	0.018	0.182	0.127	0.309
Staplers	0.368	4.049	2.209	6.258
Laparoscopic				
Ports	3.495	55.920	20.970	76.890
Scissors	1.139	18.224	6.834	25.058
Unused opened equipment				
Sutures	0.018	0.254	0.200	0.454
Packing material	0.445	–	0.445	0.445
Auto purse string clamp	25.600	–	25.600	25.600
Needle & syringe	0.069	–	0.137	0.137
Totals	–	128.268	117.714	245.982

## Discussion

### Summary of findings

The sampled procedures in this quality improvement project offer a comprehensive representation of the varied caseload covered by the general surgical department. We included cases from both Colorectal and Upper Gastrointestinal sub-specialties, encompassing a mix of acuity levels and approach types (open vs. laparoscopic). Good practice was demonstrated in areas such as reusable textiles, avoiding unnecessary catheter use, and loose skin preparation use. Small improvements were made in adoption of alcohol-based scrubbing techniques however overall uptake remained low (15%, 17%) and unnecessary non-sterile glove use was observed in >30% of procedures. Further diverse and sustained interventions may be required to influence areas requiring a change in clinical decision making. This evidence can be used to direct local initiatives towards areas with the highest potential for positive impact, and can act as a baseline from which to measure change over time.

### Findings in relation to current literature

The area offering the highest potential for carbon reduction as a single intervention going forward is the adoption of hybrid laparoscopic scissors and ports. This aligns with the observation that consumables account for 32% of operating theatre emissions (8). Hybrid laparoscopic instruments have been found to have a lower environmental impact compared to single-use equivalents, with an average reduction of 60% across 17 environmental impacts. Even when considering factors such as low instrument reuse, decontamination with separate packaging, use of fossil fuel-rich energy sources, or variations in carbon intensity during transportation, hybrid instruments still exhibited better environmental performance. Furthermore, the total financial cost of using hybrid instruments is less than half of that associated with single-use equivalents (19). Given the trend towards minimally invasive, often laparoscopic, techniques over open approaches, this is a particularly poignant area for consideration as its impact will only grow in years to come.

Another considerable saving could also be made by avoiding the opening of equipment that is subsequently discarded unused. The identification of equipment to be ready but unopened is already common practice at the time of briefing within surgical settings (20). Therefore, there may be fewer barriers to further emphasising the importance of adherence to this principle from an environmental, as well as an economical sustainability perspective compared with other changes.

Positive practices of note include the standardisation of reusable drapes and trolley covers, and the unanimous use of loose skin prep. When clinically appropriate choices exist, making these decisions at a procurement level allows the most cost-effective and sustainable choices to be embedded into clinical practice to maximum effect. For example, where supply arrangements are already established for reusables, e.g., in the case of reusable gowns, eliminating the procurement of disposable options would be a feasible step to rapidly and decisively minimise environmental impacts (21, 22). Furthermore, feedback and negotiation with manufacturers can lead to both carbon and financial savings e.g., reduction of excessive product packaging or removal of unnecessary items included within pre-prepared clinical packs (21). It is important to note however, that establishing reliable supplies and sufficient stockpiles is vital if a single procurement route is to be relied upon, as evidenced by the reduced usage of reusable gowns during the second dataset, which was due to temporary supply issues in at least 5 procedures.

While procurement decisions ensure the availability of sustainable resources, awareness and action regarding the judicious use of these resources by clinical staff remain crucial (6, 23). Healthcare professionals recognise climate change as a potential threat to human health and desire to effect positive change. Nevertheless, a high proportion perceive a lack of education and awareness regarding how climate change relates specifically to the healthcare setting, and what actions are appropriate to take, as a key barrier to implementation (21, 22).

Significant differences can be made when staff are educated and empowered to make more sustainable choices. “The Gloves are Off” campaign at Great Ormond Street Hospital (GOSH) NHS Trust in 2018 encouraged staff to make more considered risk assessments before reaching for non-sterile gloves (24). This led to a reduction of >36,000 gloves per week, equating to a saving of 21 tonnes of plastic over the subsequent year. They also observed reduced instances of dermatitis among staff, improved healthcare anxiety among patients and financial savings associated with both purchasing and disposal without any increase in hospital-acquired infections. Considering that an estimated 5.5 billion gloves are used across the NHS and social care sectors annually, scaling up such actions nationwide could have a huge potential impact (18, 24). Overuse of non-sterile gloves is a key area for potential improvement locally, however culture change is difficult. GOSH attests that it was only through varied and sustained education and awareness campaigns that they achieved these improvements (24). Furthermore, the normalisation of PPE use in all patient encounters during the COVID-19 pandemic could potentially make this shift more difficult (25).

“The Gloves are Off” campaign exemplifies how sustainable options often yield system-wide co-benefits (25–28). Sustainable development is the ability to meet the needs of the present without compromising the ability of future generations to meet their own needs (29). This encompasses economic and social factors, alongside the more widely recognised environmental aspects (28). Co-benefits are crucial for achieving a holistic definition of sustainable value, and can be a key facilitator for improving engagement. Demonstrating simultaneous financial savings or improvements in patient and staff outcomes, means changes are more likely to be embraced on a broader scale (30).

After engaging in conversations with staff members following the initial data collection period, it became evident that there was a lack of awareness and confidence in the alcohol scrub option, despite this being endorsed by NICE and its ready availability throughout operating theatres. If the remaining 73 out of 88 staff members across both datasets who used water-based scrubbing had chosen alcohol-based alternatives, an additional reduction in water usage of 1350l (equivalent to 1.87 kgCO2e) could have been achieved. As with glove use, the evolution in clinical decision making and establishment of new cultural and behavioural norms needed for successful uptake of practices such as alcohol-based scrubbing will require improved awareness, education, and clear, consistent leadership (16). Endorsement and support from professional institutions is a key facilitator in improving healthcare staff confidence in taking action on climate change (21). This emphasises the importance of visible national leadership and guidance from bodies such as NICE and the various Royal Colleges in empowering clinicians to embrace changes in the status quo, however this must be followed by dissemination and support at a local level for change to occur (6).

### Future research and action

To enable clinicians to make informed decisions regarding the environmental impacts of their practices, it is essential to improve access to supporting evidence. For several outcomes, there was insufficient information to calculate environmental impact and carbon footprint. When accessible, information was obtained through open-source databases generated by public institutions or extrapolated from previously published research. Given their direct oversight in the manufacture and supply chain of consumables, the medical technology and pharmaceutical industries are ideally positioned to provide comprehensive analysis. As such, alongside advocating for further development of reusable and responsibly sourced technology, the surgical community should emphasise the need for the generation and transparent reporting of environmental impact data going forward (30).

With a forward-looking approach, we can consider the incorporation of artificial intelligence (AI) in healthcare. Proposed sustainability-driven AI advancements include improvements in remote monitoring and telemedicine, self-care and prevention, and optimisation of resource allocation. However, the substantial carbon and resource demands of AI, particularly in its development stages, present a challenge. Although many promising use cases have been proposed, few have been successfully implemented at scale. Balancing these impacts against potential benefits is crucial, as is recognising the environmental and ethical issues linked to AI's hardware supply chains and inherent data biases. Making a careful and holistic cost-benefit analysis is vital to ensuring ethical and effective application (31–35).

## Limitations

While both datasets were representative overall, the variability in the types of procedures captured in each dataset is likely to have influenced certain outcomes. For example, the higher number of emergency cases, particularly “incision and drainage of abscess” cases likely contributed to lower numbers of scrubbed staff in the second data set. These procedures also require no primary closure, affecting absolute values for carbon footprints from sutures/staples. This contextual variation should be taken into account when considering the resulting carbons footprints presented.

Due to practical constraints, the duration of data collection periods were necessarily short. Longer periods of data collection may have enhanced procedure comparability, increasing the probability that differences in carbon footprints were attributable to interventions made in the interim period. Despite this we feel this project lays the groundwork for demonstrating a feasible method for measuring environmental impact within our operating theatres. There is potential for it to be repeated at regular time intervals, monitoring progress chronologically, or used as a baseline from which single parameters could be isolated and explored in greater depth.

All interventions implemented as part of this audit underwent validation and evaluation by the Royal College of Surgeons of England, Edinburgh, Glasgow and Ireland during creation of the Green Theatre Checklist, including consideration of safety and clinical impacts. It was not within the scope of this project to further assess causality or association of clinical outcomes due to practical and resource constraints, however it could be a useful addition to future iterations of this work.

These results are not intended to provide a comprehensive analysis of the entire patient or procedure pathway. Non-clinical carbon sources, such as operating theatre energy consumption and ventilation systems, as well as clinical anaesthetic choices contribute considerably to overall footprint but were beyond the scope of this project. Therefore, findings should be interpreted within the context of the specific clinical practices examined. Anaesthetic gas and ventilation system related emissions contribute considerably to operating theatre emission profiles and future work considering their impact will be needed in order to holistically address whole pathway emissions. The accuracy of carbon footprinting is limited by extrapolation of data calculated within similar but non-identical settings, and the boundaries set for the project.

## Conclusion

This quality improvement project highlights the potential for implementing sustainable practices within the general surgical department and establishes a foundation for continued efforts towards a more environmentally conscious and sustainable surgical environment. By focusing on areas such as the adoption of hybrid laparoscopic instruments, avoiding unnecessary equipment opening, and standardising reusable materials, significant reductions in carbon footprint and environmental impact can be achieved. The successful implementation of these practices requires improved awareness, education, leadership and a cultural shift within the healthcare community. Collaboration among professional institutions and access to supporting evidence is crucial in driving engagement and empowering clinicians on the ground to make locally relevant sustainable change happen.

## Data Availability

The raw data supporting the conclusions of this article will be made available by the authors, without undue reservation.
