# Supplementary material for: Time for change: compliance with RCS green theatre checklist—facilitators and barriers on the journey to net zero
Source: Front Surg. 2023 Oct 24;10:1260301. doi: 10.3389/fsurg.2023.1260301 (PMC10628494; doi:10.3389/fsurg.2023.1260301)
Supplement: Supplementary file 1 [file Datasheet1.pdf]

Did you know the NHS creates 5% the carbon emissions of the UK?!

That's as much as the whole of Sri Lanka, and operating theatres are a big emissions hotspot. The average operation can produce as much carbon as driving **450 miles** in a petrol car ☹ This isn't just bad for the environment, it's bad for our health too, which is why the NHS aims to be 'NetZero' by 2045! Thankfully, there's lots of easy things we can do to make practice greener, save money and improve patient outcomes.

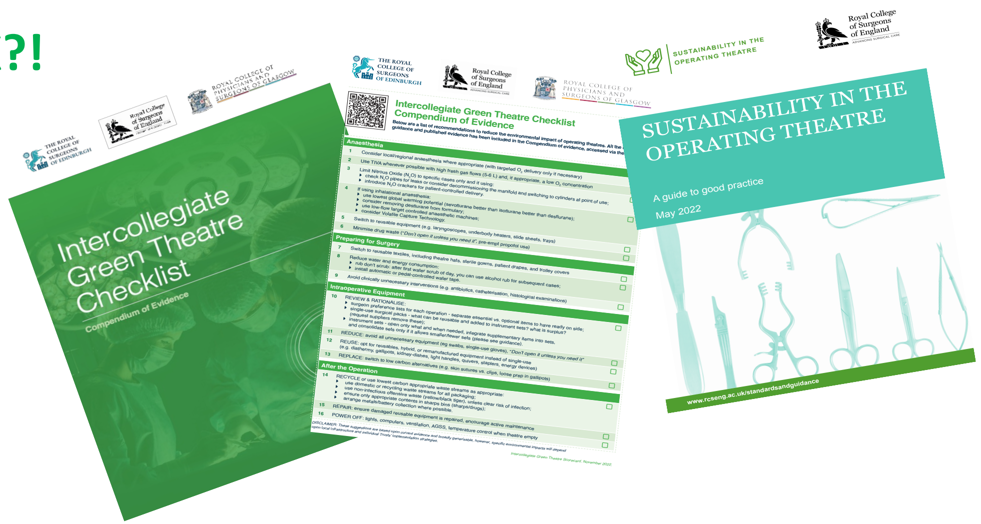

Here's 3 things *you* can change **RIGHT NOW …**

1) RUB DON'T SCRUB – after the first water-based scrub of the day, choose alcohol rub instead

A standard 3 minute surgical scrub uses 18.5L per person!!! A recent audit here in Bradford found in just 23 procedures we could have saved 1000L of water if everyone opted for alcohol rub. NICE approved, cost neutral, quicker, kinder to your skin, just as clean – what are you waiting for?!

2) DRESS TO IMPRESS – choose a reusable gown

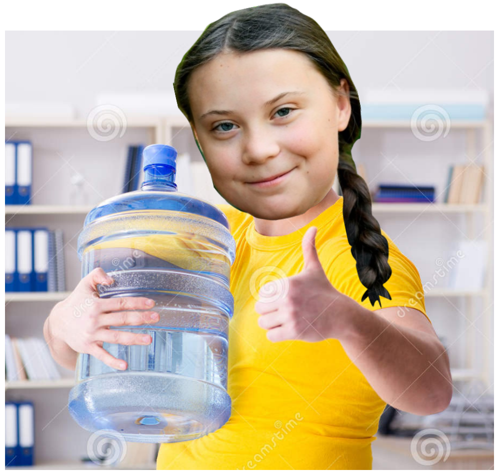

Reduce carbon emissions by 200-300%, water usage by 250-330% and solid waste by 750%. Plus you'll look Great-a (sorry)

3) THE GLOVES ARE OFF - only use non-sterile gloves when clearly indicated

Non-sterile gloves are a key piece of PPE that keep us and our patients safe, but research shows that over-use of gloves reduces proper hand washing, resulting in worse, rather than better hand hygiene. Use non-sterile gloves if there is potential for contact with mucous membranes, bodily fluids or non-intact skin, or if there are specific barrier/infection control precautions in place. In most other situations clean hands will do the job, and won't result in mountains of plastic.

Make a change - for patients, for our purse, for our planet
