# Supplementary material for: Time for change: compliance with RCS green theatre checklist—facilitators and barriers on the journey to net zero
Source: Front Surg. 2023 Oct 24;10:1260301. doi: 10.3389/fsurg.2023.1260301 (PMC10628494; doi:10.3389/fsurg.2023.1260301)
Supplement: Supplementary file 2 [file Datasheet2.pdf]

Green Surgery Audit

3 week audit of general surgical operative practice according to Intercollegiate Green Theatre Checklist.
Please complete 1 form per patient. Any patient undergoing a general surgery procedure at BRI is eligible.

* Indicates required question

1. Date *

____

Example: January 7, 2019

2. Consultant *

Mark only one oval.

- ( ) Lockwood
- ( ) Gana
- ( ) Steward
- ( ) May
- ( ) Davies
- ( ) Gokhale
- ( ) Manby
- ( ) Maude
- ( ) Halstead
- ( ) Needham
- ( ) Mosley
- ( ) Other: _______________

3. Open vs Laparoscopic

Mark only one oval.

- Open
- Laparoscopic
- Other: ______________________

4. Procedure *

____

5. Acuity

Mark only one oval.

- Elective
- Sub-acute (CEPOD)
- Acute

6. Number of re-useable gowns used

Mark only one oval.

- 0
- 1
- 2
- 3
- 4
- 5
- 6
- 7
- 8
- 9
- 10

7. Number of disposable gowns used

Mark only one oval.

- 0
- 1
- 2
- 3
- 4
- 5
- 6
- 7
- 8
- 9
- 10

8. Number of re-useable hats used

Mark only one oval.

0
1
2
3
4
5
6
7
8
9
10

9. Number of disposable hats used

Mark only one oval.

- 0
- 1
- 2
- 3
- 4
- 5
- 6
- 7
- 8
- 9
- 10

10. Re-useable trolley covers

Mark only one oval.

- Yes
- No
- Both
- Other: ____________

11. Re-usable drapes

Mark only one oval.

- Yes
- No
- Both
- Other: ____________

12. Number of staff practicing "Rub don't scrub" - after first water scrub of day, team use alcohol rub for subsequent cases

Mark only one oval.

- 0
- 1
- 2
- 3
- 4
- 5
- 6
- 7
- 8
- 9
- 10

13. If "Rub don't scrub" is not applicable i.e. first case of the day please tick below

Check all that apply.

- [ ] Not applicable

14. Catheter use?

Mark only one oval.

- Yes
- No

15. Antibiotic use?

Mark only one oval.

- Yes
- No

16. Sutures vs Staples (skin closures only)

Mark only one oval.

- Sutures
- Staples
- Other: ______________________

17. Disposable kidney dishes vs reusable kidney dishes

Mark only one oval.

- Disposible
- Reusable
- Both
- Other: ______________________

18. Prep loose in gallipots vs single use prep wands

Mark only one oval.

- Loose
- Single use prep wands
- Both
- Other: ____________________

19. Hybrid or single use laparoscopic instruments (if applicable)? Scrub staff will be able to advise

Mark only one oval.

- Hybrid
- Single Use
- Both
- Other: ____________________

20. Unnecessary non-sterile glove use? (required in anticipation of contact with a) bodily fluids b) non-intact skin c) mucous membranes or if d) specific infection control measure is in place during a patient interaction)

Mark only one oval.

- Yes
- No
- Other: ____________________

21. Non-essential/informal use of scrub gowns - if so, do staff don reusable or single use gowns?

Mark only one oval.

- Yes - Reusable
- Yes - single use
- No

22. Disposable non-essential/informal used of scrub gowns - if so, how many gowns are used

Mark only one oval.

- 0
- 1
- 2
- 3
- 4
- 5
- 6
- 7
- 8
- 9
- 10

23. Re-usable non-essential/informal used of scrub gowns - if so, how many gowns are used

Mark only one oval.

- 0
- 1
- 2
- 3
- 4
- 5
- 6
- 7
- 8
- 9
- 10

24. Please list any opened but un-used trays/equipment? e.g unopened sutures

25. Any other comments/issues/observtations

This content is neither created nor endorsed by Google.

Google Forms
